# Usefulness of bovine and porcine IVM/IVF models for reproductive toxicology

**DOI:** 10.1186/1477-7827-12-117

**Published:** 2014-11-26

**Authors:** Regiane R Santos, Eric J Schoevers, Bernard AJ Roelen

**Affiliations:** Institute for Risk Assessment Sciences, Faculty of Veterinary Medicine, Utrecht University,TD Utrecht,, P.O Box 80152, 3508 The Netherlands; Laboratory of Wild Animal Biology and Medicine, Federal University of Pará,, Rua Augusto Corrêa,Belém, CEP 66075-110 Pará Brazil; Department of Farm Animal Health, Utrecht University,, Yalelaan, 104, 3584 CM Utrecht, The Netherlands; Department of Equine Sciences, Faculty of Veterinary Medicine, Utrecht University, Yalelaan, 104, 3584 CM Utrecht, The Netherlands

**Keywords:** Female fertility, Toxicology, Bovine, Porcine, Model

## Abstract

Women presenting fertility problems are often helped by Assisted Reproductive Techniques (ART), such as *in vitro* fertilization (IVF) programs. However, in many cases the etiology of the in/subfertility remains unknown even after treatment. Although several aspects should be considered when assisting a woman with problems to conceive, a survey on the patients’ exposure to contaminants would help to understand the cause of the fertility problem, as well as to follow the patient properly during IVF. Daily exposure to toxic compounds, mainly environmental and dietary ones, may result in reproductive impairment. For instance, because affects oocyte developmental competence. Many of these compounds, natural or synthetic, are endocrine disruptors or endocrine active substances that may impair reproduction. To understand the risks and the mechanism of action of such chemicals in human cells, the use of proper *in vitro* models is essential. The present review proposes the bovine and porcine models to evaluate toxic compounds on oocyte maturation, fertilization and embryo production *in vitro*. Moreover, we discuss here the species-specific differences when mice, bovine and porcine are used as models for human.

**Electronic supplementary material:**

The online version of this article (doi:10.1186/1477-7827-12-117) contains supplementary material, which is available to authorized users.

## Background

The adverse effects caused by various natural and synthetic chemicals include impairment of both the male and the female reproductive system. Many of these effects are related to temporary or permanent actions on the endocrine system. When the mechanisms of action of these chemicals are endocrine-mediated, they may induce non-heritable defects culminating with sub/infertility, growth retardation, endocrine disorders or even death of the organism/subpopulation or its descendants [1].

Subfertility of women at reproductive age is often caused by endocrine disorders, occasionally (~10% of the cases) characterized by polycystic ovary syndrome (PCOS) [2, 3]. Although there is strong evidence that PCOS is a genetic disease, diagnoses do not always indicate the cause of the sub/infertility neither the mode of action of the causative agent [4]. It has been shown that PCOS can be correlated with exposure to the endocrine disruptor bisphenol A (BPA) [5]. Also, women with PCOS when exposed to nicotine have a higher chance to acquire other metabolic disorders [6]. Probably, many other endocrine active substances present in the environment, food, drugs or cosmetics will also cause subfertility. More than 25% of the subfertility cases are of unknown etiology and remain not diagnosed, and the affected couples attempt to achieve their parenthood experience through ART. However, knowledge on the source of subfertility, as well as the risks of oocyte defects during maturation will improve the success of IVF programs. Women exposed to a determined toxic agent may have a temporary negative effect on their oocyte quality, which may reflect on the health of the offspring.

Most information regarding reproductive toxicology has been obtained from case reports, clinical analyses and *in vivo* tests with mice and rats. The use of laboratory animals for such tests is under debate for many years [7], because *in vivo* reproductive toxicity tests require large numbers of animals. Indeed approximately 70% of all the animals used in toxicological studies are for assays involving reproductive toxicity [8]. Furthermore, rodents are not the most suitable model animals for human, especially when considering oocyte maturation and fertilization [9].

Although the *in vivo* assays should not as yet be eliminated, alternative assays including computational, integrative *in vitro* tests using embryonic stem cells and cell lines are novel approaches in reproductive toxicology [10, 11]. Available screening tests for hormone-like active compounds do not identify endocrine disruptors nor assure that a chemical will have an endocrine activity [12]. Nevertheless, such tests are useful to suggest the potential effect of certain substances. Furthermore, if the tests are integrated and projected with a clear knowledge on the main targets of reproductive toxic compounds and their involved mechanisms, the use of a large number of laboratory animals can be avoided.

Due to their complexity, not always reproductive cells such as oocytes can be mimicked by somatic cells. Moreover, oocytes and their surrounding cumulus cells have to undergo a unique process known as maturation that for the oocyte includes meiosis. Indeed, during maturation, oocytes are susceptible to epigenetic alterations that may interfere with fertilization and early embryo development [13]. Accordingly, exposure to polycyclic aromatic hydrocarbons before ART affects oocyte quality, as observed in women with a lower rate of cell division after IVF [14]. By evaluating data from different laboratories, it has been suggested that maturation of bovine oocytes can be used as a reliable model to screen toxic agents for human oocytes [15–17]. Also, the use of the porcine model has been indicated to evaluate oocyte maturation as a model for human oocytes due to some similarities between these species [18–20]. Furthermore, both bovine and porcine models can reduce the large number of laboratory animals used for reproductive testing, since oocytes can be obtained from slaughterhouse ovaries which are leftover organs when animals enter the food production chain.

The REACH (Registration, Evaluation, and Authorization of Chemicals) legislation is a European program impacting manufacturers worldwide since 2007/2008. For instance, marketing in Europe of any chemical (pure form or in a formulation) is allowed solely if data on physical/chemical properties, toxicity and environmental effects are provided. As a result, there is an enormous increase in the number of experimental animals used to test the safety of many thousands of chemicals, existing and new ones [21]. However, the adopted EU directive from 2010 focus on the protection of animals used for scientific purposes and demands a decrease in the number of animals used in research, including toxicity (drug) testing [22]. Innovative approaches have been suggested by developing a battery of tests targeting aspects of the reproductive cycle, and by integrating the approaches based on the mechanisms in cells and tissues [21]. The possibility to use slaughterhouse material for *in vitro* tests related to gametes and early embryo development appears as an important option to diminish the number of *in vivo* tests.

The aim of the present review was to evaluate the usefulness of using bovine and porcine IVM/IVF as model for reprotoxicity studies. For this, we address reproductive toxins and toxicants and their main effects on female fertility focusing on *in vitro*oocyte maturation and fertilization. Moreover, the use of oocytes from cattle and pig provides insight on the possibilities to evaluate chemicals on the *in vitro* oocyte maturation, fertilization and early embryonic development (preimplantation stage) as a model for human.

## Methods

The present review has been prepared based on a survey of data available in PubMed (1998 to June 2014), from which non-English manuscripts were excluded. The search terms were "oocyte", "*in vitro* maturation", "*in vitro* fertilization" and "toxicology", and two independent persons analyzed the data in the papers. Only studies indicating experimental procedures applying bovine and porcine cells as *in vitro* models were considered. Data from mice and human were also considered to compare with the bovine and porcine *in vitro* data. Most human data are from women submitted to ART procedures due to fertility problems. Review papers were used solely to support our introduction and discussion sections.

### Oocyte maturation, fertilization and embryo development: species differences

Oogenesis is an extremely specialized process and, depending on the species, the formation of a mature oocyte from the initial oocyte enclosed in dormant primordial follicles is completed in several weeks, as in mice [23, 24], or several months as in bovine, porcine and human [25–27]. Ovarian folliculogenesis is not detailed here, as it is well described in some recent reviews [28, 29]. When an oocyte is fully grown, it is capable of resuming meiosis, but further follicle growth is necessary to deliver an oocyte that will mature properly and give rise to an embryo after fertilization. As means of toxicological research, this is a unique event because (i) apart from that it is completed in a relatively short period of time, 14 hours in mice to 44 hours in sows, cytoplasmic and nuclear maturation require dynamic interactions that will reflect the success of fertilization [30], (ii) species-specific differences have been described during chromatin configuration at germinal vesicle stages [31, 32], and (iii) the maturation process differs among mammalian species. For example, protein synthesis is required for germinal vesicle breakdown (GVBD) in cow [33], pig [34] and human [35], but not in mice [36]. More details on the differences between large mammals and laboratory animals are available in a review from Bilodeau-Goeseels [37].

In general, after ovulation, the mature oocyte is able to be fertilized and will give rise to an embryo after a series of coordinated processes in both sperm and oocyte: spermatozoa binding to the zona pellucida, an acrosome reaction which gives the ability to the spermatozoa to penetrate the zona pellucida, binding of the spermatozoa to the plasma membrane leading to membrane depolarization and Ca^2+^ dependent events that include cortical granule exocytosis, cell cycle resumption with concomitant decreases in maturation promoting factor (MPF) and mitogen-activated protein (MAP) kinase activities, and recruitment of maternal mRNAs. After zygote formation, depending on the species, a defined number of cellular divisions take place under control of maternal mRNAs, where after embryo development will be taken over by mRNAs transcribed from the embryonic genome. Embryos with insufficient embryonic DNA transcription will cease development. Parallel to these cellular divisions, from the zygote towards the morula/blastocyst stage the embryonic genome will be demethylated and thereafter be remethylated shortly before implantation. This process of DNA methylation is indispensable for proper embryo development and protects the embryo from premature death *in utero*[38, 39].

**Figure 1:**
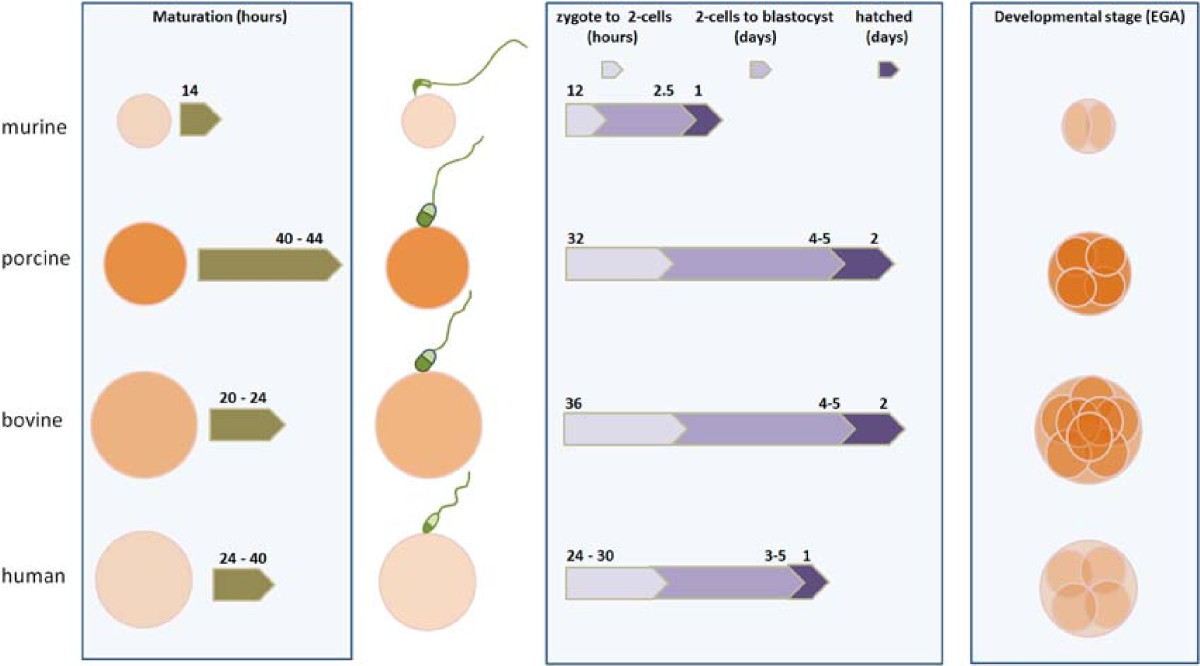
**Main differences between murine, porcine, bovine and human oocyte size at maturation, time to maturation, early embryo development and embryonic genome activation (EGA).** Color intensity of oocytes and embryos refers to lipid density. Mean size of oocytes at maturation are presented in proportion (mice ~80 μm). Average time periods of maturation and embryo development after onset of fertilization are illustrated by block arrows: oocyte maturation in hours (green arrows), transition from zygote to two-cells stage in hours (light purple arrows), transition of two-cells to blastocyst stage in days (purple arrows), and time to hatched blastocysts in days (dark purple arrows). Data were collected from *in vivo* and *in vitro* studies [34, 54–72].

Although the oocyte has conserved developmental pathways throughout evolution, phylogenetic analysis of proteins that are involved with fertilization or embryonic development revealed that human oocytes are more closely related in this respect to cow oocytes than mouse oocytes [40]. Figure 1 depicts the main similarities among murine, bovine and porcine model when compared to human. For example, oocyte diameter, the time to reach the 2-cell stage, blastocysts or hatching is similar between human, porcine and bovine, but is shorter in mice. Moreover, the time period of oocyte maturation and initial embryo development is very similar when human and bovine are compared. When the developmental stage of embryonic genome activation is observed, then human is more similar to pigs than to cattle or mice [41–43]. The morphological similarity between oocytes from women and mice is related to the cellular opacity. In these species, oocytes are translucent, while oocytes from cows are dark and from sows are very dark, due to the accumulation of lipid vesicles in the ooplasm [44]. Even though the concentration of fatty acids differ between the oocytes of the afore mentioned species, their composition is similar between these gametes [45, 46]. Lipids stored in the oocyte will function as energy source during oocyte maturation but used in a less extent during embryo development [47]. The role of the large lipid content throughout porcine embryo development remains unknown, as lipid removal from cleavage stage embryos does not seem to affect their further development to the blastocyst stage [48]. Despite of difference in oxygen consumption between bovine, mouse and porcine embryos, at the morula stage, a sharp rise of metabolic activity is recorded in all three species [44]. Murine oocytes have low amounts of fatty acids and are therefore more glucose dependent for embryo development than bovine or porcine oocytes [49]. DNA methylation results from the activity of DNA methyltransferases (DNMTs) and these proteins show a greater degree of structural similarity between human and bovine than between human and mouse [50–53].

Oocyte maturation, fertilization and subsequent embryo development involve multifaceted changes that are not easily mimicked by cell lines. Besides this, there are chronological and biochemical differences between laboratory and domestic mammals during the course of oocyte maturation and development of the embryo. Considering the feasibility to obtain ovaries from pigs and cows, the readily efficient protocols available from oocyte maturation to embryo production *in vitro*, as well as physiological and phylogenetic similarities, the use of these animal models in reproductive toxicology is encouraged [17, 73, 74]. Laboratory animals remain valuable research tools particularly humanized mice, i.e. those carrying functioning human genes, cell, tissues or organs [75]. However, oocyte maturation, fertilization and embryo development in mice cannot easily be genetically changed to be humanized; translational studies in these rodents will not always reflect the effects in human when female reproduction is evaluated.

### Exposure to toxic compounds in relation to reproductive failure

People are constantly exposed to toxic compounds present in the environment, food, drugs, and for instance cosmetics or due to occupational exposure. Exposure to several chemicals may also vary between species. For example, pigs are environmentally exposed to polychlorinated biphenyl (PCB) and dioxins, which can accumulate in fatty tissues and meat [76]. Therefore, human contamination is also related to food consumption and, as accumulated in fat, risks of exposure to new-born babies during breast-feeding is evident [77]. Human antidepressants, on the other hand, have been described as causing reproductive disorders in mice [78], while no clear effects on human fertility could be detected [79, 80]. However, such drugs can become important environmental reproductive toxicants in the aquatic environment [81]. Phthalates are environmental contaminants also used in the coating of oral medications, making the drug as a toxicant exposure source [82]. Another agent, acrylamide, is known as an occupational toxicant [17]. However, this compound can also be produced during food processing [83], including baby food and infant formulas [84], and can pass through the placenta thereby affecting fetal growth [85]. Moreover, as shown in bovine [17], acrylamide affects oocyte maturation. In the present review, we divided the toxic compounds in five main categories (environmental, food, drugs, cosmetics or occupational) for didactical reasons. For details on specific toxic compounds, Table 1 is given as reference.

**Table 1:** **Toxic compounds tested in vitro using bovine and porcine models**

***Environmental***	Species		Exposure			***Cumulus***		Oocytes			Embryos			
	Bovine	Porcine	IVM	IVF	IVC	Viability	Expansion	Viability	MII	Fertilization/Activation	2-4 cells	Blast	N nuclei	Ploidy
Anabasine [86]		86	86				86							
Atrazine [17, 87, 88]	17,87	88	17,87,88	87		17,88			17, 88	87		87		
Benzo(a)pyrene [15]	15		15	15		15			15	15				
Benzyl butyl phthalate [89]		89	89				89		89					
Cadmium [15, 17, 86]	15,17	86	15,17,86	15		15,17	86		15,17	15				
Carbedazim [15]	15		15	15		15			15	15				
4-Chloro-3-methyl phenol [89]		89	89				89		89					
Cotinine [17, 86]	17	86	17,86			17	86		17					
Cycloheximide [15, 17]	15,17		15,17	15		15,17			15,17	15				
DEHP [89, 90]	90	89	89,90				89		89,90		90	90		
Diazinon [88, 91]		88,91	88,91	91	91	88		91	88	91	91	91		
DDT [92]	92		92						92	92	92	92	92	
Fenoxaprop-ethyl [88]		88	88			88			88					
Hexachlorocyclohexane [92]	92		92						92	92	92	92	92	
Lindane [15]	15		15	15		15			15	15				
Malathion [88, 91]		88,91	88,91	91	91	88		91	88	91	91	91		
Methoxychlor [92]	92		92						92	92	92	92	92	
MEHP [17, 90, 93]	17,90,93		17,90,93			17	93		17,90,93		90	90		
Nanoparticles Au-Ag [94]		94	94				94		94					
Nicotine [17, 86, 95, 96]	17,95,96	86	17,86,95,96	95	95	17	86,95		17,95,96	95	95,96	95,96	96	96
PCB mixtures [19, 97–102]	97-99	19,100-102	19,97-101	101	102	19,99,100	19,100	19	19,97-100	19,97,98, 100,101	19,97, 98,101	19,97,98, 100-102	98,101, 102	
4-*tert*-octylphenol [103]	103		103						103	103	103	103	103	
***Food***														
Bisphenol A [89]		89	89				89		89					
α-Chaconine [104]	104		104	104	104						104	104		
Daidzein [105]		105	105				105		105	105	105	105		
Deoxynivalenol [106–108]		106-108	106-108		106	108	108		106-108	108	107,108	107,108	107,108	107,108
Flavanones [109]		109	109				109		109	109	109	109		
Genistein [15]	15		15	15		15			15	15				
α-Solanine [104]	104		104	104	104						104	104		
Solanidine-N-oxide [104]	104		104	104	104						104	104		
Zearelanone [107, 110, 111]	110,111	107	107,110,111						107,110, 111		107,110	107,110	107	107
α-zearalenol [106, 107, 112]		106,107, 112	106,107		106				106, 107		107,112	107,112	107,112	107
β-zearalenol [106, 107]		106,107							106,107		107	107	107	107
***Drugs***														
Busulfan [15]	15		15	15		15			15	15				
Diethylstilbestro l [15, 17]	15,17		15,17	15		15,17			15,17	15				
17β-Estradiol [17, 103]	17,103		17,103			17			17,103	103	103	103	103	
Ionomycin [15]	15		15	15		15			15	15				
Ketoconazole [15]	15		15	15		15			15	15				
Methyl acetoacetate [15]	15		15	15		15			15	15				
Mifepristone [15]	15		15	15		15			15	15				
Nocodazole [15, 17]	15,17		15,17	15		15,17			15,17	15				
Okadaic acid [95]	95		95				95		95					
Piperazine [17]	17		17			17			17					
Swainsonine [113]	113		113	113	113						113	113	113	
Taxol [95]	95		95				95		95					
***Cosmetics***														
Butylparaben [15]	15		15	15		15			15	15				
9-cis-Retinoic acid [114]	114		114								114	114		
Retinoic acid [17]	17		17			17			17					
***Occupational***														
Acrylamide [17]	17		17			17			17					

DDT: Dichlorodiphenyltrichloroethane; DEHP: Di-(2-ethylhexyl) phthalate; MEHP: Mono-(2-ethylhexyl) phthalate; PCB: polychlorinated biphenyl.

### Environmental toxic compounds

Although the use of some agricultural fertilizers, pesticides or animal parasitical solutions has been banned or diminished in many countries, the ability of these substances to persist in the environment (soil and water resources) presents a threat to human and animal health. This means that the negative effect of these compounds may be present longer in a population than in the environment. Moreover, for every toxic compound that has been banned or diminished, one or even more hazardous substances have come back due to adaptations in the production of new so-called less harmful metabolites. Therefore, the final threat may even increase instead of decrease or stabilize. For instance, atrazine is a 21st century herbicide which also acts as an endocrine disruptor [115] and its metabolites such as desethylatrazine [116] and cyanuric acid [117] might persist in the environment. In human, a survey quantifying biomarkers in maternal and umbilical cord serum to detect in utero exposure to pesticides showed that those agricultural contaminants affect offspring outcome and development [118]. Many reproductive dysfunctions are likely caused by environmental toxicants. To exclude this source of contamination is very difficult indeed. However, systematic studies detecting the main harmful compounds are needed to define alternatives to substitute or limit their use. From the agricultural chemicals, carbedazim has been identified as a potential endocrine disruptor as shown in a human ovarian granulosa-like tumor cell line [119]. Chlorinated pesticides like dichlorodiphenyltrichloroethane (DDT) and hexachlorobenzene (HCB) in sera from women submitted to IVF have been related to impaired embryo implantation [120]. Methoxychlor, a substitute for DDT, is a pesticide linked to female reproductive dysfunction by hypermethylation in estrogen receptors, and phosphatase and tensin homolog (PTEN) signaling, which is involved in follicular activation [121].

Plasticizers, mostly phthalates used to increase the flexibility of materials like construction products, cosmetics, medical devices or to coat medication, encompass another important group of environmental contaminants. These chemicals do not accumulate in human follicular fluid [122]. However, prenatal exposure to phthalates may lead to estrogenic or anti-androgenic effects in girls at pubertal age affecting uterine volume [123]. It can be expected that impaired steroidogenesis will lead to impaired oocyte development.

PCBs and dioxins can disturb female fertility [124] and embryo development [125, 126]. Exposure *in* utero to these compounds delayed reproductive development especially in girls [127]. There is heightened investment as well as apprehension on nanoparticle research. For example, nanoparticles containing silver are able to inhibit porcine oocyte maturation [94], but knowledge on the possible effects and endocrine interactions on human reproduction is limited [128]. The use of *in vitro* models will help to predict the risks of these compounds on oocyte maturation and subsequent fertilization and embryo development.

Nicotine and other chemicals present in cigarette smoke, e.g. anabasine, benzo(a)pyrene, cadmium and cotidine, are environmental contaminants that have not only been demonstrated to cause neoplasia but also reproductive failure. Bordel et al. [129] have shown that nicotine induces apoptosis in ovarian follicles. Different from phthalates, chemicals present in cigarette smoke can accumulate in follicular fluid. The main tobacco related compounds benzo(a)pyrene, cadmium, and cotidine for instance were found in human follicular fluid and related to fertility failure [130, 131]. Cigarette smoke also leads to reduced concentrations of anti-Müllerian hormone (AMH) in follicular fluid, which may negatively impact oocyte development [132]. More recently, it has been reported that *in utero* exposure to cigarette smoke can impair endocrine signaling in female fetuses and can inhibit the progression of early ovarian follicle development [133]. Furthermore cotinine, a nicotine metabolite, has been detected in follicular fluid from passive smokers [134, 135], which is a warning also for non-smoker women constantly exposed to cigarette smoke.

### Food toxic compounds

Together with environmental toxic compounds, exposure to food contaminants can also affect animal and human reproductive function. Food toxic agents can be of natural origin, as mycotoxins, and can be synthetic, for instance BPA. While BPA is known as an endocrine disruptor, mycotoxins have the ability to act either as endocrine disruptors or as endocrine active substances [136].

Mycotoxins like deoxynivalenol (DON), zearalenone (ZEA) and its metabolites (alpha- and beta-zearalenol) can cause reproductive failure. Although DON has been associated with gastroenteritis [137], there are reports on the reproductive effect of this mycotoxin in domestic animals [138]. Abundant knowledge is available on the estrogenic effect of ZEA in human and domestic animals [136]. However, there are many of the so-called emerging mycotoxins, e.g. enniatins, alternariol and beauverecin, that need to be screened on their action on female reproduction. For example, it is known that alternariol at high concentrations impairs progesterone synthesis in porcine granulosa cells [139]. Beauvericin might be another potential mycotoxin with reproductive effects since recently this mycotoxin was advocated to be an antitumor candidate due to its role in the inhibition of MAP kinase phosphorilation [140]. Although BPA does not accumulate in follicular fluid [122], it negatively affects human oocyte maturation *in vitro* by disturbing spindle architecture and chromosome organization [141]. However, there is a lack of data revealing if BPA affects human oviduct, placenta and pubertal development [142].

Alpha-chaconine and alpha-solanine are natural glycoalkaloids, natural pesticides, found in potatoes. These secondary metabolites are produced by potatoes when threatened by pathogens or insects. This means that injuries to some vegetables like potatoes can pose a risk to human health [143]. Besides this, most information related to female reproduction, however, is limited to studies in rats [144] and mice [145]. Phytoestrogens, with isoflavones and flavanones as the most studied are also investigated for their effects on human reproduction. Genistein is found in soybeans. Although isoflavones like genistein do not impair murine oocyte maturation [146], this phytoestrogen, as well as daidzein, can affect the development of mice reproductive organs [147]. It has been shown also that although daidzein does not affect porcine oocyte maturation, it is able to disturb progesterone secretion by cumulus cells [105]. Recently, Solak et al. [109] also showed that naringenin and 8-prenyl-naringenin impaired porcine oocyte maturation by different mechanisms. Besides these solid evidences of the risks of phytoestrogens, and the fact that human consumption of these compounds is high, epidemiological data in human are scarce and more studies using animal models are crucial as every day more phytoestrogens are recognized or included as therapeutic compounds [148].

In a sophisticated cohort study in France, Chan-Hon-Tong et al. [149] acquired detailed information on dietary exposure to potential contaminants in women before pregnancy and at the third trimester of pregnancy. Although exposure before pregnancy appeared higher than at the end of pregnancy, the fetus still can be exposed to contaminants like mycotoxins, acrylamide and PCBs. Besides, exposure before pregnancy may also impair the ability of a woman to conceive naturally, thus requiring ART.

### Drugs, cosmetics and occupational toxic compounds

Research on the endocrine effect of drugs, cosmetics and occupational toxic compounds is available but more limited. Probably because some adverse effects are already expected, like the use of antineoplasic drugs, or due the fact that exposure to occupational toxicants is already under strict regulation. However, in some developing countries children are constantly exposed to parasites, requiring a routinely based administration of drugs such as antifungals, which may act also as endocrine disruptors [150]. Maternal anemia or risks of perinatal death can be caused by helminthic infestation, which must be treated with anthelmintic drugs. But, such drugs may also have embryotoxic effects [151].

In humans a relatively constant exposure to drugs is inevitable, either by choice (contraceptives) or to treat diseases (chemotherapeutic, antiparasitic, antidepressants or antiepileptic agents) which, after chronic use, may affect fertility or embryo development [152–154]. It is well known that many chemotherapeutic agents, particularly alkylating agents, impair fertility not because of their cytotoxic characteristics but also by leading to premature ovarian failure or insufficiency, affecting oocyte reserve, activation and growth [155]. Other drugs like busulfan, diethylstilbestrol, 17β-estradiol, mifepristone, ketoconazole, piperazine, methyl acetoacetate, okadaic acid and taxol are endocrine disruptors. Although *in vitro* data in bovine and porcine show the risks that these compounds bring to oocyte maturation and fertilization, little is known about their effect on human oocyte maturation and fertilization.

Cosmetics bring concerns because of their increase in the market and augmented use by humans, including children. Among them, compounds present in UV-filters like benzophenone are endocrine disruptors that have been detected in human serum [156]. Topical use of retinoic acid has been included as a hazard for its teratogenic risk, but such exposure is too low when compared to the oral administration of vitamin A [157]. Besides, Tahaei et al. [158] showed that treatment of murine oocyte with retinoic acid during IVM has positive impact on maturation rate and subsequent embryo development after IVF.

Also, occupational toxicants have been analyzed for effects on reproduction. Acrylamide has been characterized as an occupational toxicant, once it is used to synthesize polyacrylamides, one of the compounds used in gels for electrophoresis. Due to severe legislation to protect researchers and analysts who work with this compound, most risks are avoided. However, this compound still harms human health since it is also a food toxicant. Dimethylene glycol monobutyl ether (DGBE) is present in latex paints, but is not toxic for rats after 10 weeks of exposure [159]. However, humans who by their profession may be exposed to these compounds for years might be affected. Pesticides, including insecticides and rodenticides, also act as occupational toxicants, affecting adults and children [160]. Even at low concentrations, chronic exposure to these compounds affects endocrine activity [161].

## Final remarks

Many couples apply to ART because of fertility problems and more than one fifth of the causes of sub/infertility in female are unknown. There is compelling evidence that fertility problems are related to the exposure to different sources of contaminants, among them dietary and environmental appearing as a great concern. Some of the identified chemicals can affect the oocyte already during maturation. If the oocyte does not follow an apoptotic pathway, there is still the risk that fertilization and subsequent embryo development will be somehow affected. Furthermore, when ART is necessary, patients should be advised about the environmental and dietary contaminants not only because of metabolic diseases, but also to prevent exposure to hazardous chemicals.

To identify the effect of such dietary and environmental compounds, cell lines have been used as an important screening tool. However, a cell line will not always mimic gametes during a critical phase of maturation, which requires specific biochemical changes. It is possible to apply reliable and more complex screening methods, as well as decrease the number of laboratory animals, by using bovine or porcine as models for IVM, IVF and embryo production *in vitro*. Obviously the bovine/porcine model will not replace rapid and massive screenings with cell lines, and cannot completely eliminate *in vivo* studies with rodents. Although, the research material will be obtained from healthy animals that entered the food chain and no extra facilities are necessary to house animals, bovine and porcine IVM/IVF models have certain disadvantages that should be considered when applied to test potential toxic compounds. For instance, the origin of slaughterhouse-derived materials (ovaries) is often unknown such as age, reproductive status, possible reproductive disorders or stressful conditions. To overcome this, Ovum Pick-Up (OPU) from donors with known background can provide a solution. Even though cattle oocyte development is rather similar to that of humans, in *in vivo* studies the porcine appears as a good model for transgenerational research since it is a monogastric species with a gastrointestinal tract similar to that of humans. Overweighing supposed risks of certain chemicals should be avoided. However, human beings are daily exposed to different contaminants. Even though exposure may occur at extremely low concentrations of each contaminant, chronic multi-exposure should be taken into account, and epigenetic changes affecting embryo development must be considered.

## Authors’ original submitted files for images

Below are the links to the authors’ original submitted files for images. Authors’ original file for figure 1
