# Supplementary figures and images for: Usefulness of bovine and porcine IVM/IVF models for reproductive toxicology
Source: Reprod Biol Endocrinol. 2014 Nov 26;12:117. doi: 10.1186/1477-7827-12-117 (PMC4258035; doi:10.1186/1477-7827-12-117)

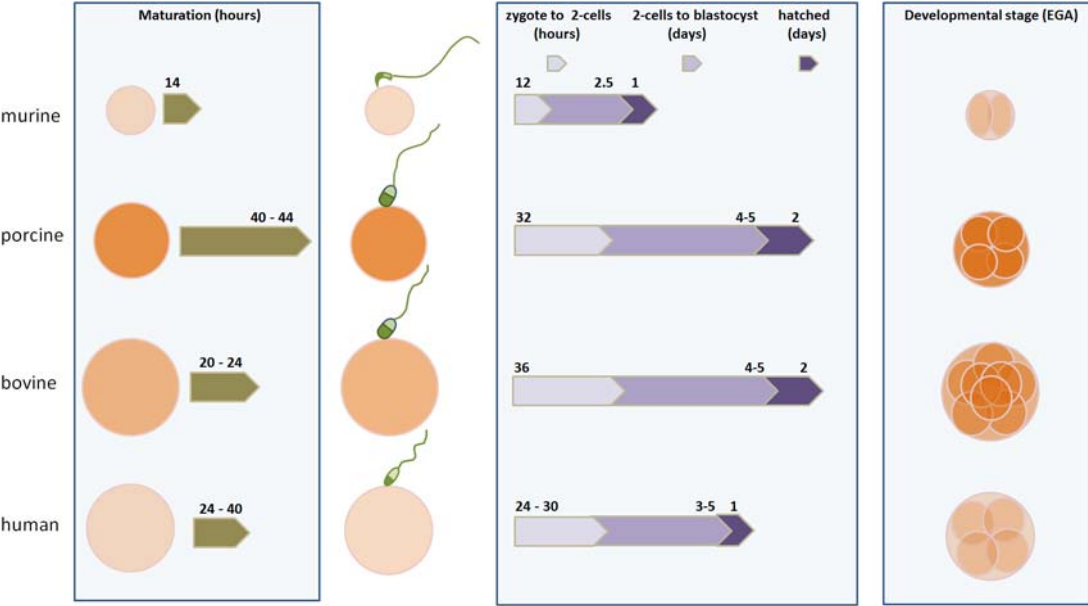

Supplement: Supplementary file 1 — Authors’ original file for figure 1 [file 12958_2014_1284_MOESM1_ESM.pdf]
